# Higher Order Architecture of Designer Peptides Forms Bioinspired 10 nm siRNA Delivery System

**DOI:** 10.1038/s41598-019-53462-1

**Published:** 2019-11-14

**Authors:** Alicia Gamboa, Selina F. Urfano, Katrina Hernandez, Deborah A. Fraser, Luladey Ayalew, Katarzyna Slowinska

**Affiliations:** 1 Department of Chemistry and Biochemistry, California State University Long Beach, 1250 Bellflower Blvd, Long Beach, California 90840 USA; 2 Department of Biological Sciences, California State University Long Beach, 1250 Bellflower Blvd, Long Beach, California 90840 USA

**Keywords:** Gene delivery, Drug delivery, Self-assembly

## Abstract

The higher-order architecture observed in biological systems, like viruses, is very effective in nucleic acid transport. The replications of this system has been attempted with both synthetic and naturally occurring polymers with mixed results. Here we describe a peptide/siRNA quaternary complex that functions as an siRNA delivery system. The rational design of a peptide assembly is inspired by the viral capsids, but not derived from them. We selected the collagen peptide (COL) to provide the structural stability and the folding framework, and hybridize it with the cell penetrating peptide (CPP) that allows for effective penetration of biological barriers. The peptide/siRNA quaternary complex forms stoichiometric, 10 nm nanoparticles, that show fast cellular uptake (<30 min), effective siRNA release, and gene silencing. The complex provides capsid-like protection for siRNA against nucleases without being immunostimulatory, or cytotoxic. Our data suggests that delivery vehicles based on synthetic quaternary structures that exhibit higher-order architecture may be effective in improving delivery and release of nucleic acid cargo.

## Introduction

The ability of siRNA to silence genes might lead to potentially transformative therapeutic strategies, but effective delivery continues to pose a significant challenge^1,2^. Various nanoparticle-based systems are recognized as the current benchmark to achieve delivery and release of siRNA^3^. Yet, the most widely used nanoparticle delivery systems, based on either viral capsules, or cationic lipid/polymer formulations, often lead to immunogenic response, induce significant levels of toxicity, tend to accumulate in liver and spleen, and are expensive to make^4–6^. Alternatively, virus-like particles prepared by reconstituted and self-assembled capsid protein have shown promising results^7^.

Cell penetrating peptides (CPP) - short, bioinspired, cationic peptides - have been designed specifically to improve efficiency of intracellular delivery^8–10^. Because of the large, permanent, positive charge, CPP’s are well suited to transport nucleic acid across the membrane. However, the CPP-based siRNA carriers often display a broad size distribution, usually between 50–250 nm, which limits their utility due to variability of cellular uptake^11–14^. Moreover, non-specific interactions with other anionic compounds, lack of adequate protection of the cargo against nucleases and proteases, and cytotoxicity, further complicate the use of CPP as carriers^13^. We have recently demonstrated that many of these challenges can be resolved by introducing architectural motifs via synthetic linking of CPP with a collagen peptide^15,16^. This resulted in the formation of a triple helical nanocarrier that effectively delivered Paclitaxel to malignant cells^17^.

The hierarchical organization of protein provides the environments that is optimized for carrying specific functions: recognition events, enzymatic selectivity, transport and protection^18^. The higher order architecture results in spatial organization that produces the nanosystems that are monodisperse and stoichiometric. The higher order architecture is difficult to accomplish with floppy polymers and peptides^19^. Thus to achieve the higher order architecture, the structural elements must be introduced on the lower level of organization to be able to propagate the order through higher levels^20^.

The folding motifs has been recently proposed as functional elements in drug delivery systems to introduce the conformational changes and supramolecular assembly as “actuators” to carry the intended biological function^21,22^. We have previously shown that the temperature induced peptide folding of hybrid peptides into higher order structure, triple helix, allows for control selection of cellular delivery^16^. The synthetic collagen peptides are also capable of forming hierarchical structures via self-assembly^23–25^. Thus we hypothesized that the assembled synthetic CPP/collagen hybrid peptide (subunits) could result in the formation of higher-order architecture, a capsid-like vehicle for RNA delivery.

The combination of CPP and collagen peptide as independent domains of a hybrid peptide resulted in the formation of a new class of hybrids that exhibit a series of unusual properties. Up to date we only studied single subunits (3 peptides folded into helix), with no hierarchical order. This work presents the higher order assembly between multiple subunits and nucleic acid.

Here we present the assembly of CPP/collagen hybrid peptide subunits that have a monodispersed size distribution, 10 nm, due to formation of stoichiometric complexes. The rigid subunits protect the siRNA located in the middle of the complex, similarly to virus’ capsids, but lack the immunostimulatory or cytotoxic characteristic typical for viruses. In addition, the assembled delivery system has the unusually small size and monodispersed character that allows better control over cellular uptake. Our results suggest that delivery vehicles based on synthetic quaternary structures that exhibit higher-order architecture may be effective in improving delivery and release of nucleic acid cargo and, in the future, display other functional elements.

## Results and Discussion

### Structural characterization of V_*h*_/siRNA nanocomplexes

**Figure 1:**
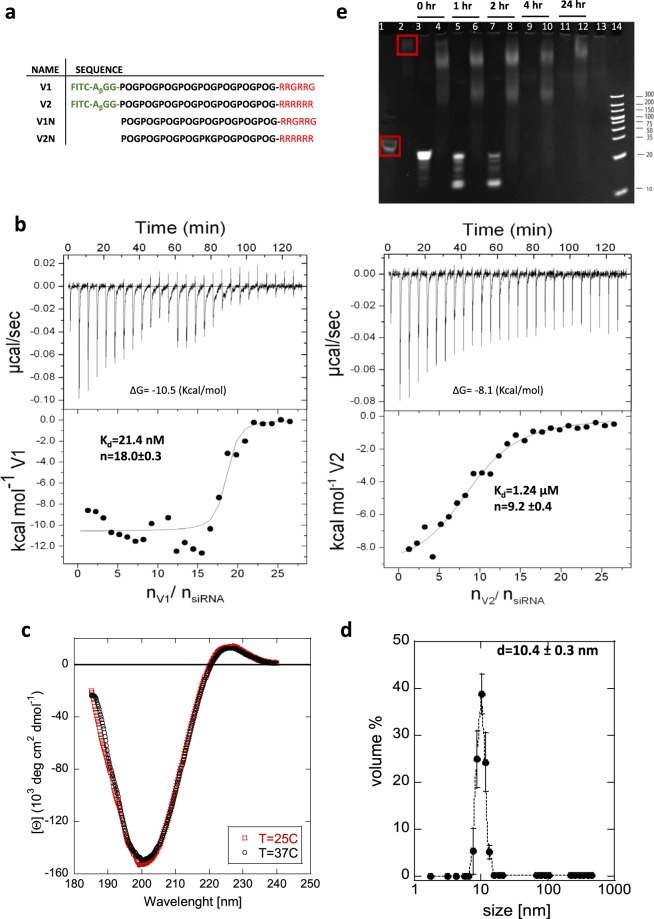
Structural characterization of peptide/siRNA complex. (**a**) Peptide sequences; “O” represents hydroxyproline, black is collagen domain, red is CPP domain, and green is FITC fluorescence tag. (**b**) ITC thermograms of V1/siRNA and V2/siRNA; calculated binding parameters were calculated assuming a single site independent model. (**c**) CD spectra of V1/siRNA complex at 25 °C and 37 °C; the same height of positive peak (λ = 220 nm) indicates folded (triple helical) conformation. (**d**) DLS size distribution by volume of V1N/siRNA complex; error bar represents standard deviation from six independent samples. (**e**) Serum stability assay: Both naked siRNA and V1N/siRNA complexes were incubated (variable times) with 10% human serum and separated in 20% native PAGE. The gel was stained with ethidium bromide. Lane 1, siRNA in MilliQ Water (red box); Lane 2, V1N/siRNA complex in MilliQ Water (red box); Lane 3, 5, 7, 9, 11 siRNA in serum; Lanes 4, 6, 8, 10, 12, V1N/siRNA complex in serum; Lane 13, serum; Lane 14, ladder. Image is a full-length gel.

We designed complexes containing the collagen domain sequence (POG)_8_ (“O” stands for hydroxyproline) and two different Cell Penetrating Peptides sequences: (RRG)_2_ (V1) or R_6_ (V2) (Fig. 1). The fluorescence reporter, FITC, was attached to the N terminus via a GGA_β_ linker, and the C terminus was amidated to block undesired interactions (Fig. 1a). The peptides are designed to exhibit triple helical conformation at 37 °C, with the transition temperature of T_m_ = 48.8 °C (V1) and T_m_ = 45.6 °C (V2) confirmed with Circular Dichroism (CD)^15^. The higher T_m_ observed for V1 results from increased stabilization of the helix by placing glycine in the preferred position (XYG) and smaller positive net charge within the helix, 12 versus 18 arginines, lowering the repulsion between the stands. Folding of the three peptides into a triple helix resulted in the formation of a rigid rod subunit (V_*h*_) with localized positive charge at the CPP domain^15^. The CPP domain is most likely unfolded.

The complex formation between the subunits V1_*h*_, or V2_*h*_, and siRNA was observed with Isothermal Titration Calorimetry (ITC). In both cases thermograms (Fig. 1b) show binding, and the plateau indicates formation of the stoichiometric complex. We used the single site binding model^12,26^ to calculate the binding ratio of 18:1 for V1/siRNA (9:1 for V2/siRNA), which corresponds to six V1_*h*_ (three V2_*h*_) subunits binding to a single siRNA. The CD spectra of the complex (Fig. 1c) show the positive peak at 224 nm corresponding to nπ* amid transition characteristic for triple helix conformation of peptide assembly. This confirms that the peptides are organized in the folded subunits within the complex at the temperatures used for structural and functional characterization of the complex, either at 25° (room) or 37° (physiological).

Both V1_*h*_, and V2_*h*_ subunits bind to siRNA with high affinity (K_d_ = 2.14 × 10^−8^ M and K_d_ = 1.24 × 10^−6^ M of V1/siRNA and V2/siRNA respectively). While we expected stronger binding between the V2 and siRNA due to a larger positive charge, the complex with V1 is stronger, likely due to cooperative effect^27^ (where six V1_*h*_ subunits participate in complex formation vs only three subunits for V2_*h*_). The thermodynamic analysis shows that the binding is enthalpy driven (Supplementary Fig. 1) and agrees well with cooperativity of long-chain macromolecules with multiple binding sites^24^. Both complexes are positively charged with the charge ratio of 1.7 (V1/siRNA) and 1.3 (V2/siRNA) which enables the complex entry into the cells (Supplementary Table 1).

The cellular uptake efficiency of drug carriers strongly depends on their size and size distribution^28^. Many CPP/cationic lipid/polymer based siRNA carriers have broad distribution of sizes^11–14^. Monodisperse systems can be formulated, but they require additional steps in sample preparation, and can be hard to accomplish^29^. Our system is based on a high affinity stoichiometric complex with higher order architecture, thus generates monodispersed nanoparticles. The dynamic light scattering (DLS) evaluation of the V1/siRNA complex showed that the complex size is 10.4 ± 0.3 nm. When complexes are modeled to be spherical, the volume distribution of 10 nm particles constitutes 98.7% of the sample (Fig. 1d, Supplementary Fig. 2). In Fig. 1d six independent measurements were averaged to plot the volume distribution (error bars reflect standard deviation of each size particle observed). The majority of cationic lipid/polymer/CPP bases nanoparticles are much larger (50–200 nm) and highly dispersed^4,11–14^. If we consider V1/siRNA complex composition based on ITC measurements, the resulting molecular weight is 76 kDa. The globular, 76 kDa protein is predicted to have a diameter of approximately 6 nm. Since a V1_*h*_ subunit is about 7.5 nm long, and we cannot assume ideal spherical shape of the complex, the measured 10.4 nnm size agrees reasonably well with theoretical predications.

The rapid degradation of siRNA by endonucleases is viewed as a major delivery barrier^2^. We have tested the protection of siRNA that is provided by the V1/siRNA complex by treatment with 10% human serum for various times and analyzed the digested samples on 20% native polyacrylamide gel (Fig. 1e). While the naked siRNA is rapidly degraded, with complete degradation after 4 h, the complex showed no sign of degradation even after 24 h. We observed broad bands in the gel, indicating the presence of the complex. The fully assembled complex carries a high net positive charge (+30, 1.7 charge ratio), thus to be able to enter the gel, it ether has to be partially disassembled, or serum-derived components are associating with the complex. The latter is supported by the lack of siRNA degradation even after extensive exposure to 10% human serum (Fig. 1e), which would not be possible for a partially disassembled complex.

To verify that the broad band represents V1/siRNA complex, we added undiluted RNase (10U/µL) to ensure degradation. Indeed, after 2 h we do not observe the complex band and the positively charged peptide is immobilized in the well (Supplementary Fig. 3). In addition, when V1/siRNA complex was treated with SDS, we observed full dissociation of the complex. Our results suggest that the siRNA is located in the central part of the complex and the peripheries are occupied by V1_*h*_ subunits. In our opinion, the architecture of the complex results in siRNA protection via the structural hindrance of the subunits.

### Cellular uptake of V_*h*_/siRNA nanocomplexes

**Figure 2:**
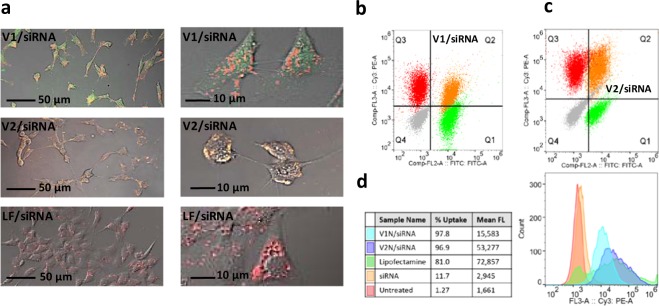
Cellular uptake by 3T3 Swiss mice fibroblasts. (**a**) Confocal microscopy image (merged all channels (DIC, FITC, AF647)) of 3T3 cells incubated with V1/siRNA(AF647), V2/siRNA(AF647), and lipofectamine(LF)/siRNA. The dye associated with V1 (green), and the dye associated with siRNA (red), are clearly separated, Pearson’s coefficient = 0.243; V2 (green) is colocalized with the siRNA (red) indicated by the yellow color, Pearson’s coefficient = 0.713 The bar represents 50 µm (first panel) and 10 µm (second panel); All controls in supplementary Fig. 4. (**b**) Multi-color flow cytometry analysis of V1/siRNA complex. Each quadrant indicates populations of cells expressing fluorescence in the respective channel: Untreated (grey) cells in Q4, V1N/siRNA(Cy3) in Cy3 channel (Q3), V1/siRNA in FITC channel (Q1), V1/siRNA(Cy3) in both Cy3 and FITC channel (Q2). (**c**) Multi-color flow cytometry analysis of V2/siRNA complex. Each quadrant indicates populations of cells expressing fluorescence in the respective channel: Untreated (grey) cells in Q4, V2N/siRNA(Cy3) in Cy3 channel (Q3), V2/siRNA in FITC channel (Q1), V2/siRNA(Cy3) in both Cy3 and FITC channel (Q2). (**d**) Flow cytometry: comparison of fluorescence intensity in the Cy3 channel of cells incubated with V1N/siRNA(Cy3) (blue), V2N/siRNA(Cy3) (purple), lipofectamine/siRNA(Cy3) (green), siRNA(Cy3) (orange), and untreated (red); The concentration of siRNA was 1 µM in all cases, except lipofectamine/siRNA(Cy3), 0.1 µm. The table in Fig. 3d lists the % cells that are Cy3+, representing uptake of siRNA; the percent of cells that uptake was measured using gated population (Supplementary Figs 5, 6).

We have shown that the V1_*h*_, or V2_*h*_ subunits are effectively taken up by 3T3 cells (>99% cell population) in a very short period of time (<30 min)^16^ (Fig. 2). To capitalize on the exceptional uptake properties of V1_*h*_ and V2_*h*_, their complexes with siRNA were prepared in the stoichiometric ratios (18:1 for V1/siRNA and 9:1 for V2/siRNA) and incubated with 3T3 cells. The peptides carried a FITC (green) tag and siRNA was modified at 3’ with an AF647 (red) tag. As controls, we used V1, V2, naked AF647-siRNA, and AF647-siRNA/lipofectamine as a benchmark transfection agent (Supplementary Fig. 4). After a two-hour incubation period of 3T3 cells with V1-siRNA and V2-siRNA the confocal images (Fig. 2a) show efficient uptake of both complexes (V1 and V2, green tag) and siRNA (red tag). There is a distinct difference between the fluorescence images of 3T3 cells after V1/siRNA or V2/siRNA uptake. While both tend to accumulate in the endosome-like bodies, the V1/siRNA complex seems to dissociate, and green and red dye are clearly separated; the V2/siRNA complex appears to stay bound (yellow color). ImageJ software was used to quantify the colocalization of FITC tagged peptides and AF647 tagged siRNA and calculate the Pearson’s coefficient. Average Pearson’s coefficient calculated for cells incubated with V1/siRNA complex is 0.243 (70 cells), and for V2/siRNA complex delivery is 0.713 (80 cells). We concluded that V1/siRNA is able to release the siRNA more effectively than V2/siRNA. This result is surprising in view of the stronger association of siRNA with V1_*h*_ (K_d_ = 21.4 nM) than V2_*h*_ (K_d_ = 1.24 µM), and may be related to polyvalency of the complexes. It has been shown that multivalent CPPs show improved endosomolytic activity^30^ (although the mechanism is unknown), and enhance endosomal escape of siRNA^31^.

To quantify cellular uptake, 3T3 cell were incubated with V1/siRNA and V2/siRNA (siRNA was modified at 3’ with a Cy3 (red) tag) and analyzed with multicolor flow cytometry (Fig. 2b–d and Supplementary Figs 5, 6). First we performed four independent measurements, were 3T3 cells were incubated with: (1) V1N/siRNA or V2N/siRNA, (2) V1N/siRNA(Cy3) or V2N/siRNA(Cy3), (3) V1/siRNA(Cy3) or V2/siRNA(Cy3), (4) V1/siRNA or V2/siRNA. Then the results of each experiment were combined in Fig. 2b,c, were the different cell populations occupy different quadrants. Over 95% cells took up these complexes (Supplementary Fig. 7), while only 81% cells took up lipofectamine/siRNA. Even though the average mean fluorescence of cells treated with lipofectamine/siRNA is higher, there was a wide variation of number of siRNA copies per cell, with a population of cells not taking up siRNA at all. This is in contrast to V1/siRNA and V2/siRNA complexes that are able to consistently deliver siRNA to all cells. The mean fluorescence intensity of delivered cellular siRNA (Cy3) is about 5 times higher when delivered as V2/siRNA than that for V1/siRNA complex, indicating that V2/siRNA is able to deliver more copies of siRNA per cell. Although V2 shows the capability to deliver more siRNA per cell, the confocal images indicate that a majority of V2/siRNA complexes do not dissociate. Whether the increased uptake of V2/siRNA can compensate for the lower cellular release of siRNA due to inhibited dissociation, and act as a functional carrier, was investigated with siRNA knockdown studies.

### Functional characterization of peptide/siRNA complex

**Figure 3:**
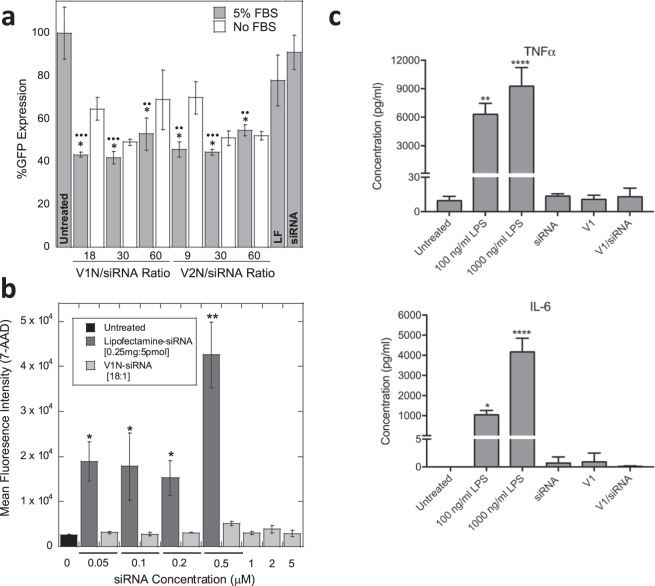
Functional characterization of peptide/siRNA complex. (**a**) GFP silencing in 3T3-GFP cells: siRNA (1 µM, except lipofectamine (LF) 0.1 µM, Supplementary Fig. 10). V1N/siRNA and V2N/siRNA treated samples were carried out in the presence (solid) and absence (pattern) of 5% FBS. Statistical differences between 3T3-GFP cell: treated (+serum) vs. untreated (•); treated with lipofectamine vs. treated with complexes (*). Increase of the peptide:siRNA ratio did not improve silencing (**b**) 7-AAD cell viability assay (flow cytometry) was performed on 3T3 cells untreated (black) or treated with either varying concentrations of V1N/siRNA (dark grey) or lipofectamine (0.25 µg:5pmol ratio) (light grey) complexes at fixed ratios after 24-hour incubation. Increase in mean fluorescence intensity indicates non-viable cells (**c**) Cytokine assays: HMDM were treated with LPS (positive control) V1, siRNA or V1/siRNA complex (1 µM siRNA) and secreted levels of inflammatory factors, TNF and IL-6, were measured by Luminex multiplex analysis. Statistical differences between untreated and treated cells were calculated by one-way ANOVA with Tukey’s multiple comparisons test. *p < 0.05, **p < 0.01, ***p < 0.001, ****p < 0.0001.

3T3 cells stably expressing GFP (Supplementary Note 1) were treated with different ratios of V1N/siRNA or V2N/siRNA (targeting GFP) (Fig. 3). The cellular fluorescent intensity of GFP was measured and compared to cells treated with lipofectamine-siRNA 72 h post transfection (Fig. 3a). The sequence selection of siRNA is described in the Supplementary Note 2. The GFP intensity measured by flow cytometry was significantly (p < 0.01 or 0.001) reduced in all samples treated with peptide/siRNA complexes, confirming that the complexes deliver and release siRNA into cells. Significantly (p < 0.05) higher knockdown efficiencies were observed for V1N/siRNA (58 ± 3%) and V2N/siRNA (56 ± 2%) than that of lipofectamine (22 ± 12%). Both V1N/siRNA and V2N/siRNA treated cells showed higher knockdown efficiencies in the presence of 5% fetal bovine serum (FBS) than in serum free conditions, indicating that healthier cells experience better gene silencing. Indirectly, the improved silencing in presence of 5% FBS also supports the results that the higher order complex architecture protects siRNA from enzymatic degradation.

The large variation of lipofectamine/siRNA knockdown efficiency (SD = 12%) reflects the problems with non-uniform siRNA delivery. Additionally, we were forced to use a lower concentration of siRNA when transporting siRNA in lipofectamine complex (0.1 µM) due to the high toxicity of lipofectamine. When an increased concentration of lipofectamine was used to transport the same concentration of siRNA as in peptide complexes (1 µM), There were no surviving cells (Supplementary Figs 8–10).

The cytotoxicity of the V1/siRNA complex was evaluated in 3T3 cells by 7-AAD (7-aminoactinomycin D) fluorescence cell viability assay (using flow cytometry) and compared to lipofectamine/siRNA (Fig. 3b). Cells incubated with V1/siRNA complex show comparable viability to the untreated control sample with no significant difference (p > 0.05), even at concentrations as high as 5 µM siRNA (90 µM V1), indicating that V1/siRNA complexes are non-toxic within the tested range. In contrast, even at the lowest-tested concentration, cells incubated with lipofectamine/siRNA (siRNA = 0.05 µM) showed a significant increase in cell toxicity (p < 0.05). At the higher concentration of siRNA (0.5 µM) a large increase in fluorescence intensity is observed indicating widespread cell death. This supports our previous observation that at 1 µM siRNA concentration transported with lipofactamine, very few cells are within the healthy gated population (based on forward and side-scatter properties). (Supplementary Fig. 10).

The proinflammatory response is often associated with siRNA delivery systems based on viral capsules or cationic lipid/polymer formulations. Macrophages readily secrete IL-6 and TNFα in response to inflammatory stimuli upon uptake^32^. The cellular uptake of V1, V2, siRNA, V1/siRNA and V2/siRNA by primary human monocyte-derived macrophages (HMDM) was measured with flow cytometry (Supplementary Fig. 11) and showed that peptide and peptide complexes were uptaken by approximately 55–75% of cells (depending on peptide), while siRNA only by about 25%. Here we tested the response of HMDM from three individual donors to V1 and V1/siRNA (Fig. 3c). Untreated macrophages secreted undetectable levels of IL-6, and low levels of TNFα (9.5 ± 3.8 pg/ml). Positive control cells stimulated with LPS showed significant dose-dependent increases in IL-6 and TNFα secretion, as expected. V1 peptide, siRNA or V1/siRNA combination, did not stimulate release of IL-6 and TNFα above basal levels, suggesting that this peptide is not immunostimulatory to macrophages.

## Conclusions

In summary, the bioinspired architecture of higher order structural elements (V1_*h*_ peptide subunits) were designed to self-assemble into V1/siRNA complexes that function as an effective siRNA delivery system. The described V1/siRNA complex is nontoxic and non-immunostimulatory. The formation of V1_*h*_ subunits self-assembled with siRNA in the core of the 10 nm nanoparticle effectively protects the siRNA from degradation. We plan to expand the structural characterization of the complexes by including other techniques (i.e. AFM and cryo-TEM). From a therapeutics perspective, it is tempting to speculate that the small size of the complex should allow easier diffusion through the ECM, promoting more effective uptake into tumor cells. At the same time, the increased size of these peptide complexes should prolong the circulation time, avoiding the rapid clearance associated with small peptides^33^.

## Methods

### Peptides

All peptides (V1, V2, V1N, V2K) were purchased from Tufts University Core Facility (Fig. 1a). V1 and V2 were modified to contain the fluorescence label conjugate FITC (fluorescein isothiocyanate) at the N-terminus linked via β-Alanine-Glycine-Gylcine (A_β_-GG) linker to prevent quenching. V1N and V2K lack the FITC reporter domain and instead contain a free amine group at N terminus (V1N) or N terminus is blocked by an acetyl group (V2K) to prevent unwanted side reactions. All peptides have blocked the C-terminus by amidation. The collagen domain consists of proline-hydroxyproline-glycine (POG)_n_ repeats, and a cell penetrating peptide domain consists of polyarginines sequences: (RRG)_2_ or (R_6_).

### Isothermal titration calorimetry (ITC)

ITC was conducted on a MicroCal Auto-iTC200 system (Malvern) with a cell volume of 400 μL. 160 μM of V1/V2 and 1 μM siRNA were prepared in ultrapure degassed Milli-Q water. Degassed Milli-Q water was used in the ITC reference cell. For each titration 1.5 μL of peptide in a pipette stirring at 250 rpm was added to siRNA solution in the sample cell at 25 °C at intervals of 60 seconds between injections for a total of 26 injections. The heat of dilution was measured by titrating V1/V2 into degassed Milli-Q water and subtracted from each sample measurement. A single site independent model was used and analyzed with Origin software.

### Circular dichroism (CD)

The V1N or N2/siRNA complex was prepared in 500 μL of Milli-Q water as described previously with final concentration 90 μM V1N and 5 μM siRNA. Circular dichroism (CD) measurements were performed using a JASCO J-810 spectropolarimeter (JASCO Inc., Easton, MD, USA) equipped with a Peltier temperature control system containing a quartz cell (path length 0.2 cm). The peptide solutions were transferred to a CD cell and equilibrated for 30 min at 25 °C or 37 °C. A scan speed of 50 nm/min was used, and four scans per sample were acquired and averaged. A reference spectrum containing deionized water was subtracted from the final peptide spectrum.

### Dynamic light scattering (DLS)

V1N-siRNA complex was prepared as described previously in 500 μL of Milli-Q water with final siRNA concentration of 1 μM. Samples were then filtered through 0.2μm filter (Whatman). Six independent DLS size measurements were performed on a Zetasizer Nano-S (Malvern) at 25 °C in a low volume disposable sizing cuvette.

### Enzymatic stability of V1/siRNA complex

V1N/siRNA complex was prepared as described previously in 18:1 ratio in MilliQ H2O so that final siRNA concentration was 14 µM. 20 µL of V1N/siRNA complex or siRNA (14 µM) was added to 20 µL of 20% human serum. Each sample contained 9 µg of siRNA in 10% human serum. Samples were incubated at 37 °C for various time points; 0, 1, 2, 4 and 24 hours and immediately frozen at −80 °C. 10 µL of sample were analyzed on 20% non-denaturing polyacrylamide gel in 1X TBE buffer. Gel was run at 200 V for 45 minutes then stained with ethidium bromide and visualized on UV transilluminator.

### NIH/3T3 cell culture

Swiss mouse fibroblast NIH/3T3 cells were purchased from the American Type Culture Collection (ATCC) and cultured according to listed specifications. Cells were cultured in T-25, T-75 flask (5.0 × 103 cells/cm^2^) in Dulbecco’s Modified Eagle Medium (DMEM 1X, Mediatech Inc) supplemented with 10% Fetal Bovine Serum (FBS, HyClone) and 0.5% Penicillin Streptomycin L-Glutamine Mixture (Pen/Strep, Lonza). Passages 3–10 were used for all experiments.

### Confocal microscopy

24 h before the experiment, 3T3 cells were seeded into MatTek dishes (No. 1.5 coverglass, 35 mm) at a cell density of 5.0 × 10^3^ cells/cm^2^ in DMEM media 10% FBS and 0.5% Pen/Strep. V1/V2-siRNA complexes were prepared as described previously in Opti-MEM 1X media. 100 µL of complex was added dropwise to the dish containing 900 L of Opti-MEM 1X media with 10% FBS so that final siRNA concentration was 1 µM. Lipofectamine 2000 was used as a positive control following standard protocol using a final siRNA concentration of 0.1 µm. After 4 hours of incubation, complex solution was removed, and the cell culture was washed with PBS three times followed by addition of DMEM 1X complete media. Cells were evaluated using a confocal microscope (Olympus FluoView).

### Generation of Stable 3T3-GFP cell line

Swiss mouse fibroblast NIH/3T3 cells (3T3) were transfected with pACGFP1-C1 plasmid following Lipofectamine 2000 protocol. Cells stably expressing the pACGFP1-C1 (GFP) plasmid were selected with DMEM 1X media containing 800 µg/mL G418 antibiotic and 10% FBS. Highly fluorescent cells were further isolated by Fluorescence Activated Cell Sorting (FACS) on a SH800Z Sony Cell Sorter. 3T3 cells stably expressing GFP were maintained with DMEM 1X media containing 500 µg/mL G418 antibiotic and 10% FBS.

### Purification of DNA plasmid

pACGFP1-C1 plasmid (Clonetech) was transformed into DH5α cells. In summary 0.5 µL of plasmid (500 ng/mL) was added to 50 µL of DH5α cells and incubated on ice for 15 minutes followed by heat shock at 42 °C for 30 seconds and incubation on ice for another 5 minutes. 950 µL of SOC broth was added and then placed on a shaker at 37 °C on low setting for 1 hour. 200 µL of cells were then spread onto LB agar plate containing Kanamycin (50 µg/mL). The plate was incubated at 37 °C overnight. The next day cells were frozen in 15% glycerol stock solution at −80 °C. pACGFP1-C1 plasmid was purified using Zyppy Plasmid Miniprep Kit (Zymogen).

### Uptake efficiency

24 h before experiment, 1 × 10^4^ 3T3 cells per well were seeded per well into a 96-well plate in DMEM 1X complete media. Peptide siRNA complexes were prepared as described previously in Opti-MEM 1X media. Cells were washed with phosphate buffer saline (PBS 1X, pH 7.4, without calcium and magnesium, Cellgro). and 50 µL of Opti-MEM media with 10% FBS was added to cells. 50 µL of complex was added to cells so that final siRNA concentration was 1 µM. Lipofectamine was used as a positive control following standard protocol using final siRNA concentration of 0.1 µM. Cells treated with V1, V2, or V1/V2-siRNA complexes were then incubated at 37 °C in a CO_2_ incubator for 2 hours. Cells incubated with siRNA or lipofectamine were incubated at 37 °C in a CO_2_ incubator for 4 hours. The cells were then washed three times with PBS 1X. Cells were then trypsinized and resuspended in 150 µL of Opti-MEM 1X media 2% FBS, transferred to 1.5 mL centrifuge tubes, and kept on ice until analysis. Samples were then examined using flow cytometry (SH800Z Sony Cell Sorter) at 10,000 events per sample. Data was analyzed on FlowJo software.

### GFP knockdown

24 h before experiment, 1 × 10^4^ 3T3 cells per well stably expressing GFP were seeded per well into a 96-well plate in DMEM 1X media with 500 µg/mL G418 and 10% FBS. Peptide siRNA complexes were prepared as described previously in Opti-MEM 1X media. 50 µL of complex was added to cells containing 50 µL of Opti-MEM media with 10% FBS so that final siRNA concentration was 1 µM. Lipofectamine was used as a positive control following standard protocol using a final siRNA concentration of 0.1 µM. Cells were then incubated at 37 °C in a CO_2_ incubator for 72 hours. Cells were washed with PBS, trypsinized, and suspended in 150 µL of Opti-MEM 1x media with 2% FBS. GFP expression levels were measured by flow cytometry (SH800Z Sony Cell Sorter) at 10,000 events per sample. FlowJo software was used to analyze results and obtain mean fluorescence intensity. %GFP expression was calculated as the relative mean fluorescence intensity to the average of untreated samples (negative control) which was defined as 100%.

### Cell viability

24 h before the experiment, 1 × 104 3T3-GFP cells per well were seeded into a 96-well plate in DMEM 1X complete media. Peptide siRNA complexes were prepared as described previously in Opti-MEM 1X media at 18:1 ratio. Cells were washed with phosphate buffer saline (PBS 1X, pH 7.4, without calcium and magnesium, Cellgro) and 50 µL of Opti-MEM media with 10% FBS was added to cells. 50 µL of complex was added to cells with final siRNA concentration of 0.05, 0.1, 0.2, 0.5, 1, 2, and 5 µM. Lipofectamine was used as a positive control following standard protocol using recommended ratio (0.25 µL of Lipofectamine per 5 pmol of siRNA) at final siRNA concentrations of 0.05, 0.1, 0.2, and 0.5 µM. Cells were then incubated at 37 °C in a CO_2_ incubator for 48 hours. The cells were then washed times with PBS 1X and supernatant and wash were collected. Cells were then trypsinized and resuspended in 150 µL of Opti-MEM 1X media 2% FBS and transferred to 1.5 mL centrifuge tubes containing the corresponding supernatant and wash. Cells were centrifuged at 1000 RPM for 5 minutes, supernatant was removed, and cells were resuspended in 100 µL of Opti-MEM 1X media 2% FBS. 7-AAD (7-amino-actinomycin D) viability staining solution (eBioscience) was added according to recommended specifications (0.5 µL of dye was added to each sample containing 100,000 cells) and incubated for 5 minutes on ice in the dark. Samples were then examined using flow cytometry (SH800Z Sony Cell Sorter) at 10,000 events per sample. Data was analyzed using FlowJo software.

### Human monocyte-derived macrophages isolation and culture

Human blood samples for isolation of monocytes, were collected with informed consent from healthy donors into EDTA by a certified phlebotomist according to the guidelines and approval of California University Long Beach Institutional Review Board. Human monocytes from 3 individual donors were isolated using the EasySep Direct Human Monocyte Isolation Kit according to the manufacturer’s instructions (StemCell). Cell purity was determined using the Scepter cell analyzer (EMD Millipore, Darmstadt, Germany) and monocyte populations used were greater than 90% pure. Isolated monocytes were cultured for 8 days in RPMI1640, 10% FCS, 2mM L-Glutamine and 1% penicillin/streptomycin containing 25 ng/ml rhM-CSF (Peprotech, Rocky Hill, NJ) to stimulate differentiation into human monocyte-derived macrophages (HMDM).

### Cytokine assays

HMDM were added to tissue culture treated plates at 5 × 10^5^ cells/mL in HL-1 (Lonza, Walkersville, MD) serum-free defined media. Cells were cultured for 18 hr at 37 °C in 5% CO_2_ alone or with added ultrapure LPS (Invivogen, San Diego, CA), V1, V2, siRNA or complexes of peptide/siRNA. Preparations of peptide and siRNA were prepared under sterile conditions. Supernatants were harvested, and centrifuged to remove cellular debris. Secreted levels of TNFα and IL-6 were quantified by Luminex multiplex analysis (Millipore) according to the manufacturer’s protocol. Data are average responses of three individual donors run in technical triplicates.

## Supplementary information

Suplementary Information
