# Supplementary material for: Higher Order Architecture of Designer Peptides Forms Bioinspired 10 nm siRNA Delivery System
Source: Sci Rep. 2019 Nov 14;9:16875. doi: 10.1038/s41598-019-53462-1 (PMC6856157; doi:10.1038/s41598-019-53462-1)
Supplement: Supplementary file 1 — Suplementary Information [file 41598_2019_53462_MOESM1_ESM.docx]

Supplementary Information

**Higher Order Architecture of Designer Peptides Forms Bioinspired 10 nm siRNA Delivery System**

Alicia Gamboa^1^, Selina F. Urfano^1^, Katrina Hernandez^1^, Deborah A. Fraser^2^, Luladey Ayalew^1^, Katarzyna Slowinska^1^*

^1^Department of Chemistry and Biochemistry, California State University Long Beach, 1250 Bellflower Blvd, Long Beach, California 90840

^2^Department of Biological Sciences, California State University Long Beach, 1250 Bellflower Blvd, Long Beach, California 90840

Contents

1. Supplementary Figures………………………………………………………………S2

Figure 1: **Thermodynamic Profile**

Figure 2: **DLS correlation Coefficient**

Figure 3: **Enzymatic degradation of siRNA**

Figure 4: **Cellular uptake and controls**

Figure 5: **Cytometry analysis of 3T3 cells: V1**

Figure 6: **Flow Cytometry analysis of 3T3 cells: V2**

Figure 7: **Uptake Analysis of 3T3 Cells by Flow Cytometry**

Figure 8: **Cell Viability: lipofectamine**

Figure 9: **Cell Viability: V1**

Figure 10: **Toxicity of lipofectamine**

Figure 11: **HMDM uptake**

2. Supplementary Table………………………………………………………………..S11

Table 1: **Charge ratio of peptide-siRNA complexes.**

3. Supplementary Notes………………………………………………………………..S12

Note 1: **Target Gene Selection**

Note 2: **siRNA Selection**

**Supplementary Figure 1. Thermodynamic Profile**

Isothermal Titration Calorimetry was used to compare V1 and V2 binding interactions with siRNA and thermodynamic parameters were calculated using Single Site Binding Model.

**Supplementary Figure 2. DLS correlation Coefficient**

DLS raw correlation coefficient and size distribution by volume of V1N/siRNA complex for six independent measurments.


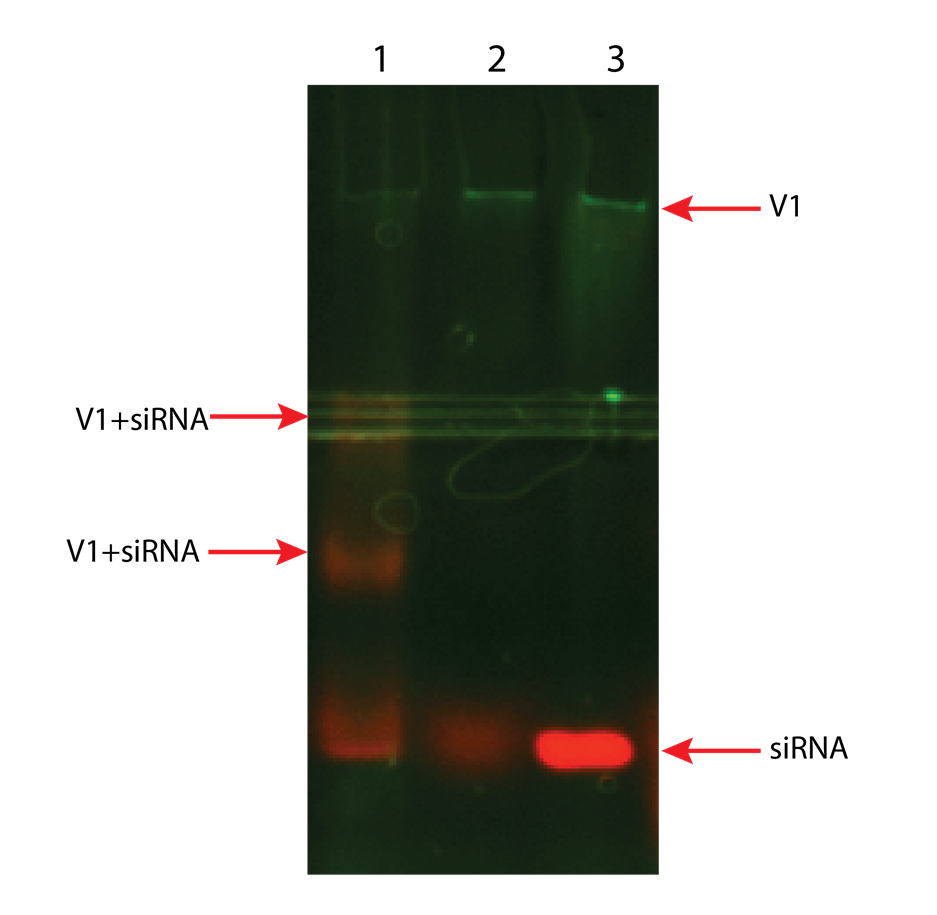


**Supplementary Figure 3. Enzymatic degradation of siRNA**

V1-siRNA samples were treated with serum, RNase or SDS and run on 20% native PAGE gel and imaged using chemiluminescence. Lane 1, V1-siRNA in 100% serum; Lane 2, V1-siRNA after 2-hour incubation with 10U/µL RNase; Lane 3, V1-siRNA complex treated with SDS. V1 contains FITC tag (green) and siRNA contains Cy3 tag (red).

**Supplementary Figure 4. Cellular uptake and controls**

Confocal microscopy images (20x) of 3T3 Swiss mice fibroblast cells: Comparing uptake and colocalization of untreated, siRNA-AF647 (1µM), V2-FITC (9µM), V1-FITC(18µM), lipofectamine-siRNA-AF647 (0.1µM), V2-FITC-siRNA-AF647 (9µM:1µM), and V1-FITC-siRNA-AF647 (18µM:1µM). Untreated, siRNA-AF647, and lipofectamine were incubated for 4 hours. V1, V2, V1-siRNA, and V2-siRNA were incubated for 2 hours. Bar represents 50µm.


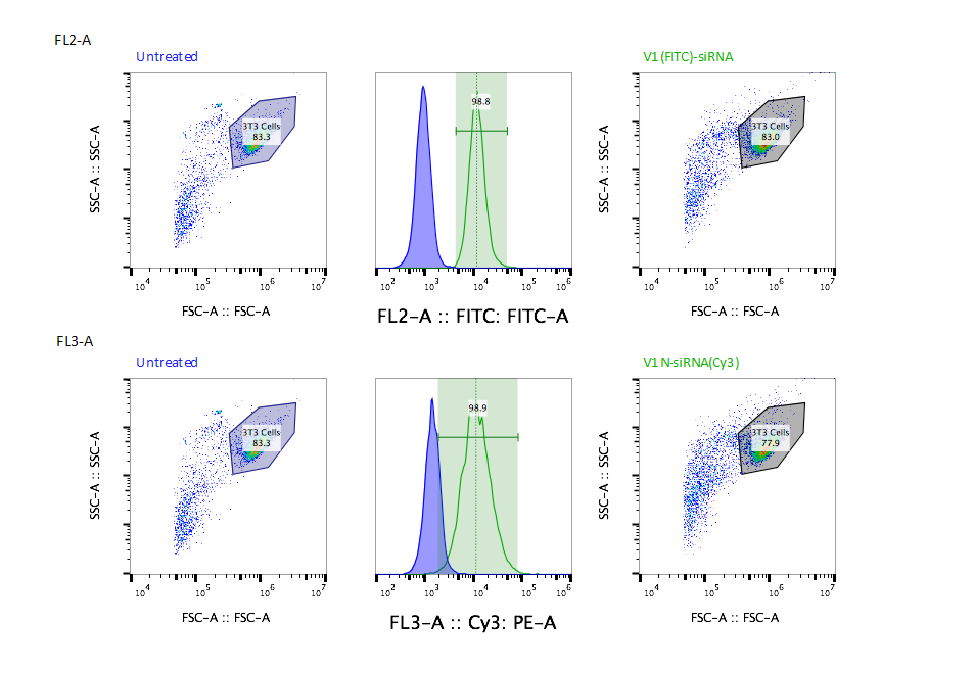


**Supplementary Figure 5. Flow Cytometry analysis of 3T3 cells: V1**

Compensation panel for V1-siRNA complex. Left panel shows size of population of untreated cells. Middle panel shows fluorescence intensity of cells in respective channels, FITC (top) or Cy3 (bottom). Untreated cells are shown in purple, V1(FTIC)-siRNA (top), and V1N-siRNA(Cy3) (bottom) are shown in green. Right panel shows population of cells treated with V1(FITC)-siRNA (top) and V1N-siRNA(Cy3) (bottom).


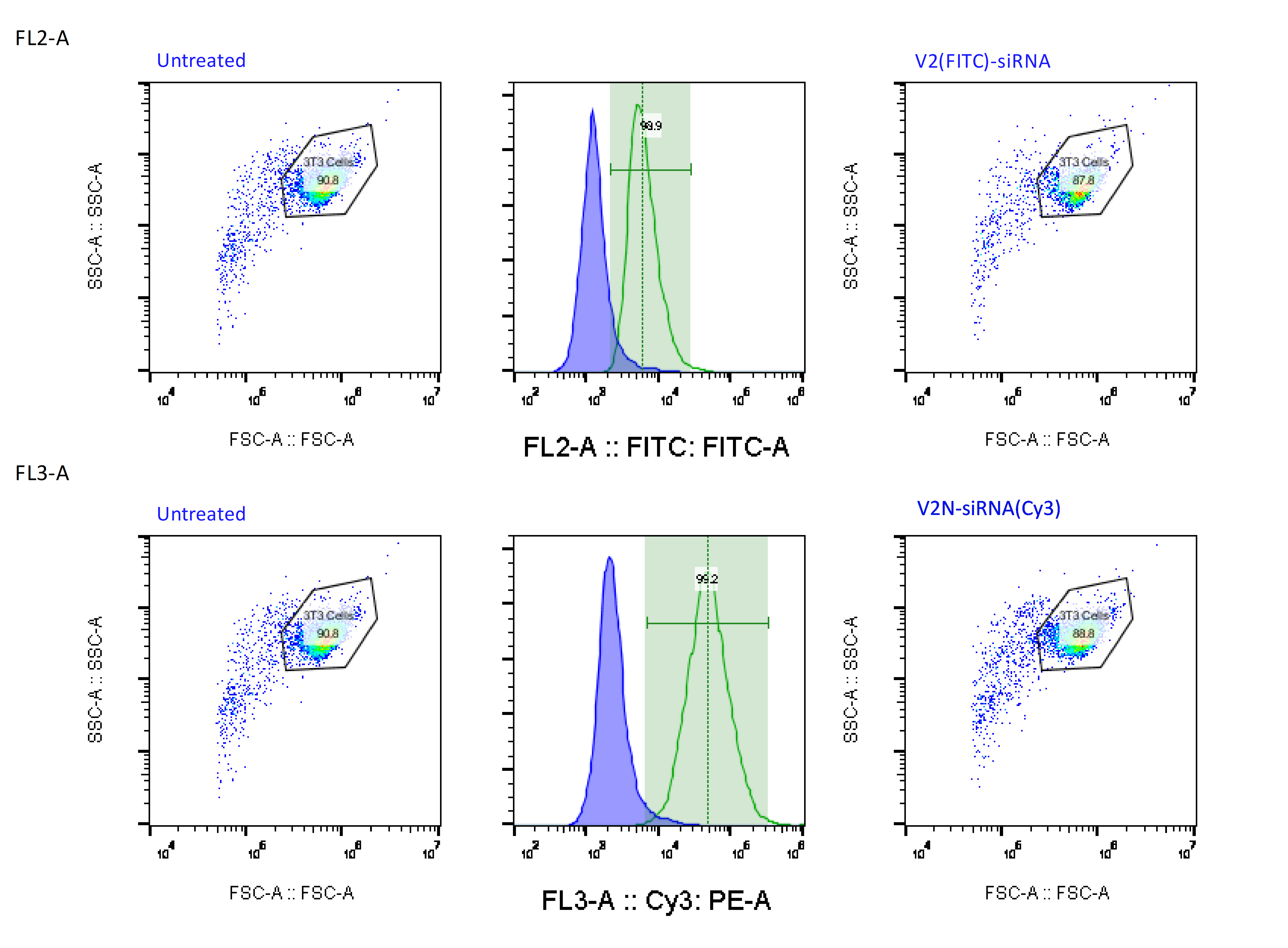


**Supplementary Figure 6. Flow Cytometry analysis of 3T3 cells: V2**

Compensation panel for V2-siRNA complex. Left panel shows size of population of untreated cells. Middle panel shows fluorescence intensity of cells in respective channels, FITC (top) or Cy3 (bottom) (purple). Untreated cells are shown in purple, V2(FTIC)-siRNA (top), and V2N-siRNA(Cy3) (bottom) are shown in green. Right panel shows population of cells treated with V2(FITC)-siRNA (top) and V2N-siRNA(Cy3) (bottom).

**Supplementary Figure 7. Uptake Analysis of 3T3 Cells by Flow Cytometry**

The siRNA uptake efficiency is measured based on fluorescence intensity observed in Cy3 channel and corresponded to cells from Q3 in Figure 2b and 2c.

**Supplementary Figure 8. Cell Viability: lipofectamine**

7-AAD Assay (flow cytometry). Analysis of cell viability on gated cell population of lipofectamine treated samples to untreated control. Everything outside of the selected polygon gate is considered to be debris. The mean fluorescence detected in the FL4-A channel is color coded where blue indicates no fluorescence and red indicates high fluorescence. The increased number of yellow, orange, and red dots indicates increased number of non-viable cells.

**Supplementary Figure 9. Cell Viability: V1**

7-AAD assay (flow cytometry). Analysis of cell viability on gated cell population of V1N-siRNA complexes in comparison to untreated control. Everything outside of the selected polygon gate is considered to be debris. The mean fluorescence detected in the FL4-A channel is color coded where blue indicates no fluorescence and red indicates high fluorescence. The increased number of yellow, orange, and red dotes indicates increased number of non-viable cells.

**Supplementary Figure 10: Toxicity of lipofectamine**

Flow cytometry compensation panel for 3T3-GFP cells. (**a**) healthy gated population of untreated 3T3-GFP cells. Middle panel shows fluorescence intensity of cells in FITC (green) channels. (**b**). 3T3-GFP cells treated with lipofectamine/siRNA (1µM). Middle panel shows fluorescence intensity of cells in FITC (green) channels. Right panel shows diminished fluorescence intensity of 3T3-GFP cells in FITC (green) channels. The decrease in fluorescence intensity is associated with the catastrophic cell death, not the GFP silencing.

**Supplementary Figure 11. HMDM uptake**

Cellular uptake of V1, V2 siRNA, V1/siRNA and V2/siRNA by macrophages (HMDM) measured with flow cytometry. The incubation time is 3h at 37°C. The star indicates monitoring of FITC (green) channel; all other data was acquired by monitoring Cy3 (red) channel. The concentration of siRNA was constant (1µM) , V1 was 18µM, and V2 was 9 µM, according to the stoichiometry of complexes.

**Supplemental Table 1.**

Charge ratio of peptide-siRNA complexes.

**Supplementary Note 1: Target Gene Selection**

Green fluorescent protein from Aequorea coerulescens (GFP) was chosen as the target protein to measure RNAi mediated silencing because the quenching of the fluorescence due to silencing is easy to detect. Flow cytometry allows for accurate quantitation of GFP in each individual cell and selection of the population expressing only high levels of GFP and production of a more uniform cell population. Flow cytometry can also detect cells with varying viabilities and identification of apoptotic cells. Thus, this technique can help select only healthy cell populations and simultaneous monitor cytotoxic effects of transfection reagents. GFP has minimal to no effects on cell viability, therefore it can be assumed that any toxic effects to cells is most likely due to the transfection reagent. siRNA mediated knockdown of GFP can also be monitored through flow cytometry, which is a more sensitive detection method compared to western blotting or PCR since it allows for evaluation of individual cells*

* Ho, H. et al. Flow cytometry for assessment of the efficacy of siRNA. *Cytometry Part A,* **69**, 1054-1061 (2006).

**Supplementary Note 2: siRNA Selection**

siRNA sequence is an important consideration for effective knockdown experiments. Initially a general GFP siRNA, Silencer GFP (eGFP), was used in two forms: with and without fluorescence tag Cyanine 3 (Cy3) or Alexafluor 647 (AF647). Although this siRNA was successfully complexed and delivered by all V1, V2, and Lipofectamine (imaged by red tagged siRNA), it was ineffective in silencing the GFP that was being expressed in 3T3 cells. In order to achieve an effective knockdown, the siDESIGN center from Dharmacon was used to generate a

functional siRNA specific to the GFP plasmid expressed in the 3T3 cells. The siRNA was designed to target the open reading frame (ORF) since this region is usually the most conserved and less polymorphic. The GC content was chosen to be approximately 50% in order for the antisense strand to efficiently separate from the sense strand and bind to its target with high affinity. Dharmacon uses an algorithm that provides the likelihood of successful silencing of 50 siRNA sequences and presents them from highest to lowest score. The siRNA sequence with the highest score with probability of successful silencing to be >90% was chosen (Table below, Dharmacon) and used for GFP knockdowns studies, although knockdown success is only guaranteed (100%) if a pool of 3 siRNA’s is purchased. Since the goal of this work is to design effective siRNA delivery method we used single siRNA, not a pool, and measure relative success of the silencing achieved with that siRNA. In addition, this experimental design has better control over what silencer is delivered. The used siRNA sequences are listed below.

| siRNA Sequence | Experiment | Source |
| --- | --- | --- |
| 5’ /AF647/CAAGCUGACCCUGAAGUUCtt 3’  5’ GAACUUCAGGGUCAGCUUcc 3’ | Confocal Microscopy | Ambion *Silencer* GFP (eGFP) |
| 5’ /Cy3/CAAGCUGACCCUGAAGUUCtt 3’  5’ GAACUUCAGGGUCAGCUUcc 3’ | Flow Cytometry; Uptake Efficiency/Serum Stability | Ambion *Silencer* GFP (eGFP) |
| 5’ AAGCUGACCCUGAAGUUCtt 3’  5’ GAACUUCAGGGUCAGCUUcc 3’ | ITC/Polarized Light Microscopy | Ambion *Silencer* GFP (eGFP) |
| 5' GCAAUAAGAUGGAGUACAAuu 3'  5' UUGUACUCCAUCUUAUUGCuu 3' | GFP Knockdown/DLS/  Serum Stability | Dharmacon |
